# 1. The British and Foreign Medico-Chirurgical Review. 2. The London Lancet, for January Arid February. 3. The True Pathological Nature of Cholera, and an Infallible Method of Treating It. By Geo. Stuart Hawthorne, M. D. 4. On the Cryptogamous Origin of Malarious and Epidemic Fevers. By J. K. Mitchell, A.M., M.D., Professor of Practical Medicine in Jefferson Medical College

**Published:** 1849-04

**Authors:** 


### REVIEWS.

ART. IV.—1. *The British and Foreign Medico-Chirurgical Review.*
2. *The London Lancet*, for January and February.
3. *The true Pathological Nature of Cholera, and an Infallible Method of treating it.* By GEORGE STUART HAWTHORNE, M.D.
4. *On the Cryptogamous Origin of Malarious and Epidemic Fevers.* By J. K. MITCHELL, A.M., M.D., Professor of Practical Medicine in Jefferson Medical College. Philadelphia: Lea and Blanchard. 1849.

The return of Cholera to this continent, in the exhibitions of its power and devastations in the northern and southern emporiums, has awakened a renewed interest in the minds of medical men and of the great public as to the characteristics of the present visitation, and the results of an enlarged experience in the treatment of this formidable agent of death. Our object in the present paper is to give as concise a view as possible, of all the interesting features of cholera: its history, its character, and its treatment.

We believe that the disease is intimately connected with malaria; we are sure that it is always found in connection with a certain conjunction of solar heat, moisture, and decaying vegetable matter. The facts on this subject are so multitudinous, so clear, precise, and unmistakeable in all cases where *all* the circumstances, attendant upon epidemic cholera, are well known, that it is in accordance with Newton's great laws of philosophy for us to assume the existence of the causes of malarial formation for every point where cholera has appeared. These laws are:

"*First.* We are to admit no more causes of natural things than such as are both true and sufficient to explain their appearances.

"*Second.* To the same natural effects we must, as far as possible, assign the same causes.

"*Fourth.* In experimental philosophy, we are to look upon propositions collected by general induction from phenomena as accurately, or very nearly true, notwithstanding any contrary hypothesis that may be imagined, till such time as other phenomena occur, by which they may either be made more accurate, or liable to exceptions."

These rules cannot be too indelibly impressed upon the minds of medical investigators. If they are faithfully applied, they cannot fail to guide the inquirer to the paths of truth. In a survey of all the recorded attacks of cholera, in every part of the earth, we have faithfully applied these laws for philosophising, and in our judgment nothing can be more certain than the fact that cholera is universally a malarious disease. We have not adopted this as a favorite hypothesis; we went to the study of epidemic cholera not to prove a theory, but to learn the truth. While investigating cholera in a particular place, we faithfully examined everything that had been said about the attack at that place; then we examined the topography of the locality, and applied the facts of physical geography to each and every investigation. The result was that the malarious origin of the disease was clearly and firmly established. Meteorology is another branch of medical science that is fast rising into great importance, and it is destined to shed a flood of light upon a great number of medical questions, and to none will it impart more than to the habitudes of malaria. This science lent us no little aid in our investigations respecting cholera.

We cannot here dwell upon the proofs of the malarious origin of cholera, but our readers may rest assured that the doctrine rests upon immutable truth, and we do not know a fact that stands in opposition to it. But before leaving this part of our subject, we beg leave to urge upon our readers the great importance of a diligent pursuit in everything that can throw light upon the subject of malaria, and upon epidemic diseases. There are certainly no finer, nor wider fields for the medical inquirer than these, and they have not yet been exhausted. In addition to a thorough understanding of malaria itself, the medical investigator should make the truths of physical geography and the facts of meteorology as familiar companions, as the letters of the alphabet are to him. These solid and invaluable sources of medical knowledge are too often neglected; — they cannot be too well impressed upon the mind of him who wishes to arrive at truth, that priceless jewel in all earthly matters.

When cholera was making its approaches to Europe, a general suspicion existed that it was contagious. The most rigid quarantines ever established sprung up in consequence of this suspicion, and a torrent of error swept over Europe as the *avant courier* of the epidemic. Among the prominent sources of error, was the idea, most industriously circulated, that cholera was a new disease, one that had never been heard of before its appearance at Jessore in 1817. One of the results of this present error was the search after a new cause for this new epidemic, and the shortest mode of solving all the difficulties of its etiology was to pronounce it contagious. Enlightened and philosophichal investigations, however, established the fact that cholera is not by any means a new pestilence, but an old and familiar form of disease. Sydenham described it among the summer di eases of England, and some of his cases of cholera morbus were unquestionably, by his description, cases of what is now called Asiatic cholera. Mr. Corbyn, a very intelligent surgeon of the Bengal establishment, published a work on cholera in 1832, and, upon a long experience in the disease, states the following conclusive fact: "In 1814, I was myself eye-witness to the destructive operation of this disease (cholera) on board the ship *Mangles*, on which I embarked for India. *We had been at sea about two months, when it burst forth with awful violence.* During the short period of six weeks, sixty-four bodies were thrown overboard, and four men died one after another just as we had cast anchor in Table Bay." This extract is full of instructive truth. Here was a vessel which left an *English port* in 1814, and, after being out at sea two months, was visited with this awful pestilence *three years before the appearance of the disease at Jessore.* Sixty-eight persons died of cholera on the ship before she reached the Cape. It is evident that there was no contagion in this case; the cause of cholera on the *Mangles* was formed in the hold of the vessel, and the common opinion, even among medical men, that cholera never was heard of before its outbreak at Jessore in 1817, is thus shown to be very erroneous. And at this point we beg leave to call the attention of the reader to an impressive fact that has urged itself forcibly upon our attention in the investigation of epidemic diseases on ships at sea. In some instances yellow fever has almost depopulated a ship, in the midst of the ocean, and when the ship was examined in the hold it was reported perfectly clean, and free from all sources of malaria. These things were very mysterious until light was thrown upon them by the case of the ship Pergamus. That vessel suffered severely with yellow fever, and upon examination her hold was pronounced clean, until what are called the *limber* boards were torn up; then a mass of putrefying vegetable matter was found in sufficient quantity to account for the awful distemper. These facts show the necessity of a thorough examination in all attempts to scrutinise the source of disease. The confined hold of a vessel may make malaria, and hold it even when there is wintry weather upon the decks, and there are situations on land that may do the same, as we shall see hereafter. But, to return, we have established the fact that Sydenham describes cholera, in his works, among the English summer diseases. He says that it is as certain to appear in summer as swallows are. We have also proved, by Mr. Corbyn, that this awful distemper showed itself on the ship *Mangles* in 1814, three years before its supposed origin at Jessore.

The idea that cholera appeared in England in 1832, for the first time, is almost as generally entertained, as that the disease originated in Jessore in 1817. But Dr. Thackrah, of Leeds, has conclusively demonstrated that the disease prevailed extensively at Leeds in 1825. That well informed gentleman gives some valuable meteorological facts, as the precursors and attendants upon the disease in 1825, and if all medical men would as carefully record such truths as these, we should have no difficulty in reaching true conclusions respecting the cause of cholera. Dr. Thackrah says: "In May, 1825, three-eighths more rain fell than the usual quantity; and the wind was east nearly the whole month. In June the weather was showery, and, on some days, *particularly sultry;* on the 12th the thermometer reached 87°; the wind was variable; but principally west. In July the weather was sultry, barometer high, winds variable, and the quantity of rain particularly small, not exceeding one-fifth the usual quantity. About the 3d of August, the drought which had prevailed for several weeks, was relieved by refreshing showers; during this month the quantity of rain exceeded the average, the weather was sultry, the barometer low, and wind generally west." Now let the reader mark the exhibition of the disease, and compare it with the above meteorological facts. Dr. Thackrah says: "In May and June, cholera appeared under its usual form. A great number of individuals were attacked, but few died. *The symptoms were such as appeared in the common cholera, therefore need not be specified.* But in July and August the disease assumed a more aggravated character. The mode of attack, and the progress of the disease, differed from the preceding cases, and the result was more fatal."

"A person would be suddenly attacked at work, or on returning to his home, or in bed, with pain in the abdomen, particularly around the umbilicus, purging almost immediately ensued, first, of common feculent matter, but the intestinal contents being soon evacuated, there followed watery or mucous stools, with excruciating tormina before each evacuation, continual and urgent calls to stool, and painful tenesmus. If appropriate remedies were at this stage promptly applied, relief was generally obtained. Some cases, however, baffled every endeavor to mitigate them; while others admitted of temporary relief; but soon the disease would reappear, and with augmented vigor. *The most alarming prostration of strength would come on, with vomiting and purging of large quantities of fluid, resembling gruel or barley water, occasionally of the color and appearance of a solution of mashed peas: cramps of the legs, and sometimes of the arms; intense spasmodic contractions of the abdominal muscles; pulse quick, and often imperceptible; skin cold and clammy, sometimes confined to the extremities, at others extending over the whole surface; countenance vacant; face of a most ghastly aspect; eyes sunk in the orbits and motionless, the eyelids and around of a purple, or more correctly of a leaden hue; lips purple; the face, even of a young and plump individual, became contracted like to the face of a toothless old man in a few hours; the patient would lie on his back with*

*the extremities extended, the arms lying by his side; the skin of the fingers corrugated, and the cellular tissue beneath exceedingly dense, in short, it was like the hand of the dead; respiration slow, laboring, and imperfect. Brandy, opium, ammonia, and every stimulant that could be suggested, in conjunction with artificial heat, and friction over the body would fail to resuscitate. In the course of a day, or often of a few hours, death would result.*

*"In other cases the disease would suddenly appear in all its malignancy, without any premonitory symptoms. The patient would sink in two or three hours under accumulated agonies, without even the slightest reaction taking place."*

Dr. Ayre also describes an epidemic cholera in England in 1817. His publication was made in 1818, and there is no difference between the cases he then described, and any that have shown themselves since. Dr. Ayre described the symptoms in 1818 of the epidemic of the previous year, in England.

These conclusive facts show clearly that the common opinion, and uniform representation, that cholera showed itself at Sunderland, England, in 1832, for the first time, are great errors. The representation that the disease displayed its powers at Jessore, in 1817, for the first time in the world's history, clothes the distemper with a mysterious and marvellous character that is inconsistent with its true nature. The fact we have given from Mr. Corbyn proves, that the disease broke out, in 1814, in an English vessel, on its outward bound voyage from an English port, after the vessel had been out at sea two months. And Mr. Corbyn is competent authority on the subject, for he had an extensive acquaintance with the disease in India, up to the publication of his book in 1832. The pamphlet of Mr. Thackrah, describing cholera in Leeds, England, in 1825, not only completely dispels the notion that cholera was a new disease in England, in 1832, but also establishes its malarial origin, by the meteorological facts we have quoted from the pamphlet, the topography of the localities where it prevailed in Leeds; and by the fact that other recognized forms of malarial disease were present with cholera, and that the latter kept pace with the former. If cholera in its epidemic form has ever been, in any one case, the result of malaria, that is the universal cause of it, according to those rules of philosophising which were propounded by Newton, and which all subsequent philosophy has confirmed.

We have shown thus clearly that cholera prevailed on the ship *Mangles* three years before the point of time almost universally set forth for its origin, and that it was in Leeds, England, seven years before the time generally named for its first appearance in Great Britain. But we shall now ascend higher. In a letter, written by Dr. Paisley at Madras, dated 12th November, 1774, we have an accurate account of cholera, and he says it often prevailed epidemically. *Sonnerat*, whose travels in India occupied the time between 1774 and 1781, gives a very fair account of epidemic cholera, on the Coormandel coast. Between Cherigam and Pondicherry, about sixty thousand persons perished. The investigations of the Madras board, go to establish the fact that "*Cholera maintained its influence, with little apparent interruption, from a very remote period, down to a date comparatively modern.*" A committee of British medical officers at Mauritius, report that, "from the statements of several individuals, some of whom belong to the medical profession, it does appear, that a disease, most strongly resembling in its symptoms, progress, and termination, that now under consideration, (the cholera of 1819), did for sometime prevail in this colony in the year 1775."

In 1781 cholera prevailed extensively as an epidemic at Gangam. On the 22d of March, 1781, a division of

British troops, marching to join Sir Eyre Coot's army, was attacked at Gangam. The cholera assailed the soldiers severely. "Men previously in perfect health dropt down by dozens; and those even less severely affected were generally dead or past recovery within less than an hour. The spasms of the extremities and trunk were dreadful: and distressing vomiting and purging were present in all." It prevailed in many other parts of India in the year 1781.

In 1782, Curtis noticed the disease in India, and describes outbreaks of the disease from 1782 up to 1787, in various parts of India. Curtis describes the disease with great accuracy. He observed that it prevailed in malarial periods, and that cold weather stopped its ravages.

Girdleston describes cholera at Madras in 1781. In 1783, cholera destroyed 20,000 people assembled at a festival. From the investigations of the Bengal board, it is plain that there are medical reports of the existence of cholera in India from 1769 down to the present time, and we have no doubt, it has existed ever since malaria began to show its powers on man.

In addition to the testimony of Mr. Corbyn, on the existence of cholera on the ship *Mangles* in 1814, we have the statement of Mr. J. Tryllie that the disease exhibited itself in India in 1814. Mr. Cruikshanks also states its extensive prevalence in India in 1814. And here is an important fact, which throws much light upon the difficulty of tracing cholera prior to 1817. The fact is this: although it is evident that cholera prevailed extensively in 1814, "*no trace of it is found in the public records*, and but for the incidental remark of Mr. Duncan, made five years after the occurrence, and we had been able most fortunately to refer to Mr. Cruikshanks, the medical returns of the corps never could have led to a knowledge" that the cholera had existed in 1814, among the British troops. "Hence," says the *Medico-Chirurgical Review*, "though cholera very rarely appears in the sick returns of former times, *it is by no means to be thence inferred that it did not then exist.*" This is a very important fact to be borne in mind, in tracing the history of cholera. Here, when we know that cholera prevailed severely among the British troops in 1814, we are unable to show the fact from the medical returns of the very troops that had the disease; but we have no doubt it has often prevailed in Europe under the names of "Black Death," and "Sweating Sickness," though, from the imperfect reports of medical men of that day, we are unable to distinguish clearly, what they referred to in their statements respecting the distempers of the 13th, 14th, and 15th centuries. There are evident traces of cholera in some of the accounts of the "sweating sickness" at Marseilles.

We have said enough now to disrobe cholera of those mysterious and marvellous garments, in which it was supposed to be enveloped, by the suspicion that it made its advent into the world in 1817, at Jessore. It was reasonable to suppose that, if it then appeared for the first time, it must be dependent on some cause that had never been in existence before, because if the cause of cholera had previously existed, its effects must have shown themselves. But we have established the fact that it prevailed as an epidemic frequently from 1760 down to the present time, and we present a cogent fact, which shows that even the silence of medical reporters on the subject, is no proof of its non-existence. We repeat, that there is no reason to doubt that cholera has prevailed in the world as long as cholera morbus has, and we know that that is no new disease.

The question is often asked, why is it that cholera does not prevail in every season suitable for the production of malaria? but we might as well ask why any other form of malarial disease does not prevail every season. Meteorology and natural philosophy, will yet make important revelations on these points; solar heat, one of the most important elements in the production of malaria, is much better understood now than it was a few years ago, and there are large fields of inquiry yet remaining in the subject. And when meteorology is universally studied by observation, over the whole earth, under all the modifications of climate, we shall begin to understand the minute details of etiology. To make ourselves correctly understood, let us take an illustration. The annual mean temperature of the earth is the same year after year, and has probably always been so; but in some years, particular localities have unusual elevation of temperature and an unusual amount of rain, and these localities, which are usually healthy, may, under these unusual circumstances, become seats of pestilence. In 1832, Lexington, Kentucky, which is ordinarily one of the healthiest cities on this continent, had sporadic cases of cholera in malarial spots, but in 1833 she had one of the most awful visitations of the pestilence that has been known in this country, and meteorology explains why it was so. There was an elevation of temperature, and almost a continuous fall of rain for a month, utterly unlike anything of the kind ever known there before; these, acting upon the flat, undrained part of the town, which part had been the receptacle of the filth of the town for some years, produced the pestilence. The epidemic broke out in the part of the town we have named, and spent a large part of its violence in the localities influenced by that flat, undrained part of the city. The phenomena of cholera, at Lexington, in 1833 would be entirely unaccountable, but for the meteorological facts we have named. The disease showed itself too in New Orleans in the depth of the winter months of December and January, but meteorology explains this phenomenon. The thermometer stood at summer heat, and there was a great amount of rain, thus were formed the three elements of malaria: solar heat, moisture, and decaying vegetable matter, and a frightful epidemic cholera thus raged in New Orleans in the winter months. A knowledge of the laws of malaria, and of the peculiarities of Louisville enabled us to make two predictions on the subject of cholera, both of which were fulfilled to the letter. In 1833, while cholera was ravaging various parts of Kentucky, we predicted, in a letter to Professor Yandell, that Louisville would have no more of the disease than the sporadic cases which were then occurring. There had been no cause for general malaria; hence the sporadic nature of the disease in this city. Again, when the pestilence showed itself in New Orleans, in December last, great alarm was felt in this city, and in a public paper, we risked everything upon the declaration that there was no reason to fear for Louisville; that she could not possibly have the disease through the winter, not only because malaria could not form in the winter, but because the fall had not been productive of malaria to poison the system of persons, so as to exhibit those occasional cases of malarial disease that occur in winter. It requires a close acquaintance with the habitudes of malaria to enable one to speak thus confidently, but such predictions may be safely adventured, when they are based upon a full knowledge of the subject. We repeat, that both these predictions, made against the earnest entreaty of friends, were fully borne out by time, the great arbiter. These facts are referred to in proof of the malarious origin of cholera, and to show how accurately the laws of malaria have been obeyed by cholera.

It may be thought that something should be said of the substantive character of malaria, and men sometimes argue against its existence, because of the ignorance that exists on this point. But we do not know anything of the substantive character of the contagion of small pox; we know its laws, and its effects, but beyond that we have no knowledge of the subject. And we know the laws of malaria, and its effects, and there our knowledge ends. A variety of attempts have been made to discover the essence of malaria; it is supposed by some to resemble carbonic acid gas; others have thought they detected sulphuretted hydrogen in malarious air; but neither hypothesis is worthy of a moment's consideration. Professor J. K. Mitchell, in one of the works at the head of this article, has propounded and defended the theory of the connection of fungi productions with malarious disease. According to this theory we may look for an ague in a mushroom, and for a pestilence in a crop of cryptogamous plants! The theory is sustained by Professor Mitchell with a great deal of ingenuity, but not with much logical force. He does not state malarial laws accurately; is not correct in his topographical difficulties, nor does he account, nor can he on his fungi theory, for the immunity of persons, in malarious localities, living in the upper stories of buildings. The down of the thistle is easily wafted by the air above the utmost height of dwellings, and fungi should be easily propagated in the same way. These facts are fatal to the theory. The facts that malaria is the product of a certain conjunction of certain properties of heat, moisture, and vegetable matter, and that it produces a great variety of diseases, are not defective in any one point, they are harmonious in all their features, when studied under the lights of Physical Geography and of Meteorology. We shall apply the laws when we come to speak of sanitary measures.

One of the most astonishing features of cholera, when viewed in connection with its malarial origin, is found in its appearance, in a few places, in winter, and as we have seen no effort made to examine this point fully, we shall bestow some attention upon it. The fact is usually stated in much stronger terms than is warranted by the truth. Some of those gentlemen who have undertaken the grave duties of the historian, and many theorists, have spoken of cholera invasions in such terms as lead the reader to suppose that cholera has raged as fiercely in winter as in summer. But this is far from being true. Cholera is emphatically a summer and autumnal disease, and its winter attacks are only exceptions to a general rule. We shall now attempt to seek an explanation of these phenomena.

When malarial diseases show themselves in winter, they show nothing of that general action that they manifest in summer, as a general rule. Indeed, this is almost universally the case. They are usually the result of the action of malaria formed late in the fall, and it lies latent until some exciting cause develops it. No fact is more firmly established, than that there is frequently a long time between the incubation of malaria, and the manifestation of its power. This was noticed extensively in what was called the Walcheren disease. That was a violent form of pestilence, which carried off large numbers of the British army in a few weeks, and the remains of the regiments were sent to England. The disease continued to attack the men, who went home apparently in good health, eight and nine months after their arrival in England, when no such disease had ever been known in the localities where these cases occurred. And the attacks were confined to those who had been on the Island of Walcheren. Here, then, a poison of great power laid dormant in the system for many months.

Another striking instance of this fact is recorded in the Medico-Chirurgical Review. A British regiment was stationed in India, at a point somewhat notorious for yellow fever. A frightful attack of yellow fever manifested itself, and committed great ravages. After a considerable portion of the regiment was destroyed, the remnant of the troops was ordered to a remarkably healthy point, occupied by another regiment, where malarial forms of disease were unknown. Cases of yellow fever continued to occur four months after the removal, among the soldiers of the affected regiment, while there was nothing of the kind in the regiment that previously occupied these healthy quarters. This conclusively establishes the fact that the most violent forms of malarial poison may lie in the human system perfectly quiescent for a great length of time. An analogous fact is found in one of the animal poisons—that productive of hydrophobia. From forty days, to twenty-four months elapse, in this disease, between the inception of the poison, and the development of hydrophobia; and this fact shows that there is no absolute relation between the power of a poison, and the celerity of its attack.

It is evident, then, that malaria may be received into the system, lie dormant some months, and then develope itself with great power. These facts are in accordance with the observations of many of the best minds that have adorned medical science. Dr. James Johnson, of the Medico-Chirurgical Review has borne, in his work on "Change of Air," decisive testimony to the truth of these statements, and extended observation, and experience have abundantly confirmed these views.

It is evident that, if malaria forms with great rapidity, under the proper proportions of solar heat, moisture and vegetable matter, a violent pestilence may spring up, and commit its ravages with great rapidity, but long after the violence has passed away, and the epidemic has ceased, cases of the disease continue to occur, for weeks and months, among those who did not yield to the epidemic influence.

This has been universally observed to be the case in all malarial epidemics. It was so at Lexington in 1833, and at New Orleans during last winter. The disease was violent in the latter place for a few weeks, its violence ceased, as we predicted it would, under the influence of cold weather, and it afterwards exhibited the feature we have named—those in whom the cause was latent, continued to fall under its power for weeks afterwards. Here, indeed, is a clear manifestation of the truth we are endeavoring to illustrate. When the weather turned cold in New Orleans, the disease so far abated, that instead of three and four hundred cases a day, there were three and four a day, and the exhibition of cholera disease finally passed off to an occasional case, and these phenomena were occurring in cold weather. While the weather in December and January was hot, the cause was active and violent; as soon as the cold weather destroyed the *unconfined* malaria, the cholera was left to attack those who had imbibed the cause of it while it was active, but in whom it remained quiescent for weeks, and then showed itself in cold weather. The same state of things often displays itself when the malaria forms late in the autumn. The cause is formed and imbibed in hot weather, and displays its effects in cold weather. Thus we have malarial disease in winter. But let it be borne in mind that malarial diseases rarely ever prevail extensively in winter, the attacks are sparse and far apart.

We should do injustice, however, to this part of the subject if we were to omit all reference to the fact that an epidemic from malarial causes may appear in winter. An instance is recorded by Dr. Cooke, which sheds so much light on this subject, that we shall quote its details. Dr. Cooke says, that it is obvious that if individuals may, after imbibing malaria, have it latent in them for sometime, and then have it developed in cold weather, the same thing may happen to the whole community, and he gives the following case in proof. He says:

"In 1748, a man built a dam across the central valley of Bethlehem township, in Connecticut, in order to kill the timber on a piece of ground containing about fifty acres. The ground continued covered until the spring of 1750. The water was then let off, and was followed by a pestilence, which carried off a number of the most robust inhabitants. * * * Ten years after, the same low ground was again overflowed; the water was let off, and another wasting sickness began in the month of November."

In fact, the universal testimony of the best and most accurate medical writers, in various years, and in various climates, to the appearance of malarial diseases through the winter and spring, after an autumn suitable for the production of malaria, and of their being confined in winter to the districts that are malarial in summer and autumn, is so decisive, that if cholera had not shown itself occasionally in cold weather there would have been reason to suspect that it was not malarial.

Let us now turn our attention to a few facts respecting the cholera of winter.

Among the most prominent of these winter cases is the recent development of cholera at Tooting, England. It shows in strong colors, the combined effects of malaria, bad food, and the want of ventilation, and this has a bearing upon the sanitary regulations, which we shall recommend. It also throws light upon a most important point respecting winter malaria. The doctrine on this subject is, that cold weather puts an end to the production of malaria, and, as a general rule, it is true. But it has been too universally applied. It is very certain that malaria, in confined sewers, ditches, etc., may resist the effects of cold weather, as we shall see when we come to speak of Edinburgh and London. The cholera at TOOTING, in January, 1849, throws much light on this subject, and we proceed to a detail of the facts. A Mr. Drouett, of Tooting, hired from the Poor Law Commissioners of a number of work-houses, a large number of pauper children, for whose board and lodging he received a weekly allowance, and he made a feeble allowance of food to the children. The children were miserably lodged and poorly fed. From the Coroner's inquest we learn, that fifteen hundred children were in the establishment of Mr. Drouett, and they were kept on miserable fare. One gentleman reported to the Commissioners, that he saw one hundred potatos cut up for the children. Not one of these potatos was fit to eat—they were black with disease. No salt was allowed in the food of the children, and they got no bread. Almost their sole diet was liquids. The results are matters of interest to medical men. Upon comparing twenty-five Welsh school children with twenty-five of these eleemosynary boarders at Tooting, the following results were found. The aggregate age of the Welsh children was 259 years, and of those at Tooting 255 years. The weight of the Welsh children was 1516 lbs. 12 oz., of those of Tooting, 1339 lbs. 8 oz., showing a difference in favor of the Welsh boys of 177 lbs. 4 ounces. The measurement across the thick part of the arms showed, in the Welsh boys, 179½ inches; in the others, 161¾ inches. The measurement of the abdomen, however, makes the most important revelations. The boys of Tooting measured, in the aggregate, 595 inches around the abdomen, while the Welsh children measured only 567½ inches; all the facts thus show, that fluid diet tutumifies the abdomen, and causes the limbs to waste away.

The lodging wards measured 196,785 cubic feet, from which, if the space occupied by the beds and children be deducted, it leaves 186,870 feet of respirable air, being only 136 feet to each child, or as much as might be contained in a box five feet two inches square. In an unventilated room this would be disastrous to health. The total number of children allotted to the beds was 1301. Instead of each child having at the rate of 266 cubic feet of air, they had within a fraction of 144 only. This, without a current through the rooms, would last only eight hours, as each expiration of breath contains four per cent. of carbonic acid gas, while ten per cent. is sufficient to destroy life.

Here then we have miserable food, and miserably small and ill-ventilated lodgings, the favorite concomitants of cholera, for these children at Tooting. Let us now trace the malaria to its sources.

The Coroner's inquest, on this calamity at Tooting was conducted by Mr. Wakley, the editor of the London Lancet. Upon examining the premises, it was found that the dormitories of the elder girls were in two old family mansions, facing the main road in the village of Lower Tooting, and a portion of these mansions was occupied by Mr. Drouett and his officers. The boys were lodged in a number of detached outbuildings, extending on the right or *north eastern* side of the play-grounds, their entire length. Beyond these, on the same side are a number of cottages, used as dormitories for the elder boys, and these cottages stand over a stagnant ditch, which passes along the end of the grounds, and these lodgings were the most unhealthy of the buildings. In some detached buildings in the same yard, were the dormitories for the younger children. These dormitories were immediately over the lower rooms, which were 10½ feet in height. The drains and ditches in the vicinity of Drouett's establishment were carefully examined by the Inspectors, and the medical men present. It was found that at the end of the grounds, the cottage sleeping apartments were built over a ditch, which formerly ran from Tooting to Wandsworth, but which has been turned, and now passes in a north eastern direction towards the Surrey Lunatic Asylum. After passing along the end of the Asylum grounds, for a short space, the ditch becomes wider, and returns again towards Tooting.—*This ditch had been cleaned out six weeks previously by a number of the larger boys of Mr. Drouett's establishment, and the filth had been deposited on the bank,* to be used as manure for the adjoining land.

In a field adjoining Drouett's place, on the farm of a Mr. Rawlings, was found a stagnant and filthy ditch of extraordinary dimensions. This ditch is situated in a *north eastern* direction from Drouett's, and is connected with an immense tank, in which all the refuse of the Lunatic Asylum is thrown. This ditch is three feet deep, from eighteen to twenty feet wide, and was nearly full of stagnant filth when examined, and emitted a most offensive odor. The ditch had been dammed up to collect and retain the filth, to be used as manure for the farm of Mr. Rawlings.

Here, then, were abundant sources of malaria, and it was in a position to be held for winter use. It was imprisoned in these ditches, but it did not *begin* its influences in January. It exhibited them, then, for the first time, but we have already given conclusive proof that a long time may elapse between the incubation and the development of the disease. Before the Coroner's inquest, a number of highly respectable physicians pronounced the disease to be cholera. Mr. Grainger, whose fame is well known in this country, said that the cases were worse than those at Glasgow, where he had been spending sometime, under the orders of the government. The worst features, of what is called Asiatic cholera, were present in these cases at Tooting, but the patients were remarkably exempt from cramps, an absence that has been often noticed. Among the witnesses on this occasion was a Mr. Robinson, surgeon of the St. Pancras work-house. He was very lucid and precise in his testimony. He declared that though the nature of the food given to the children, and the scantiness of their clothing were the predisposing causes of the epidemic, undoubtedly the main cause of the outbreak was malaria, blown, by the *north easterly* winds, from the ditches to the buildings.

We have entered into the details of this outbreak at Tooting, on account of their deeply interesting character. It is a recent case, and was rigidly scrutinized by Mr. Wakley, and the medical gentlemen whose services were called in requisition on account of the severity of the visitation. In the course of a few days 229 of the children were attacked with cholera, and, up to the 8th of January, 52 of them died, and the attacks and mortality continued up to the 15th of January, the date of the last London Herald we have received. We have condensed from two numbers of that paper, the details we have given. These details are of the profoundest interest to the medical man. They show most impressively the evils and disasters attendant upon bad food, improper clothing, ill-ventilated lodging rooms, and malaria. The poor, in all parts of the earth, have been the special victims to cholera, and their want of good food and good dwellings undoubtedly has much to do with marking them out particularly for the ravages of the disease. They need the counsel and sustaining care of the intelligent physician, and never does he exercise his profession for a holier or loftier purpose than in preventing the calamities of disease from assailing the hovels of those who are wretched enough even in health.

Let us, however, trace these wintry cases of cholera farther. In February, 1832, cholera showed itself in Glasgow, in Scotland. We take its incidents from a publication made by a gentleman, who was not only a contagionist, but an ultra one of that school. Our readers will remember that we said, that malarial diseases make no great outbreak, as a general rule, in winter, but occur in scattered cases, and are spread over a considerable space of time, when compared to a summer visitation. Thus, when cholera visited New Orleans in the summer of 1835, several thousand persons were attacked in the space of a few weeks, but in its wintry visitation at Glasgow, in 1832, there were only 140 cases in six weeks; from this time it declined to the sixteenth week, in which only five cases were reported. "*A large proportion of the cases occurred in lanes, closes, and houses remarkable for domestic and public filth;*" we are quoting from an ultra-contagionist now. "The persons attacked in these localities were, for the most part, ill-fed, badly clothed, of dissipated habits, residing in densely crowded, imperfectly ventilated dwellings."

We now turn to Edinburgh in 1832, and the history of cholera in that city strikingly illustrates what we have said of its wintry character. A good deal of malarial disease had shown itself in the autumn of 1831. Diarrhea and typhus fever were prevalent. On the 27th of January the first cases of cholera appeared. Three persons were seized on that day, and three more on the 28th. From this time to the 6th of February, no other cases occurred; up to the 15th, but six other cases occurred; on the 16th and 17th of February, seven; and between this and the 22d, but a single case appeared. In March the disease ceased. And this is often called "the prevalence of cholera in Edinburgh, in winter!" Why, we have had more than this number of violent cases of fever and ague this winter, in our own practice, among persons who were not the subjects of the disease the preceding autumn. In the depth of winter some of these cases occurred, of a most malignant type, one of which was invariably ushered in with violent convulsions. Of their malarious origin scarcely any physician can have a doubt.

Dr. Stark, of Edinburg, has published a very graphic picture of the disease in Edinburgh, in the winter of 1832; and he states that there was not a case of cholera that occurred out of the usual track of fever. It was entirely confined even in the winter, not only to the malarious districts, but to the old and familiar malarial localities. If the disease did not depend upon malaria, why did it attack no one but those under the influence of that agent of disease? And a host of testimony to the same point, bears witness to this truth in every spot of the earth, where cholera has made any exhibition of itself in winter. Dr. Stark's minute details of the drainage, sewerage, filth, etc., of the localities in Edinburgh, where the cholera made its assaults are very interesting and satisfactory, but we cannot detail them.

There are forms of malarial disease that show themselves in winter, as, indeed, they all do except those depending on exceedingly high temperature. The French Academy have established the malarious origin of Plague beyond disputation, yet it exhibits sometimes the wintry peculiarities of cholera. In 1834, in Egypt, the deaths by plague, in December were 109; in January, 1835, 151; February, 821; March, 4,329; April, 1,897; May, 321; June, 21 cases. In June the land is usually overflowed, and the water stops malaria and plague. We find repeated notices, in cholera reports, of the prevalence of diarrhea, dysentery, and other forms of malarial disease, in connection with cholera, both of summer and winter. Some malarial fevers are ushered in with diarrhea. Thus, Dr. Paxton, of Rugby, gives a history of a fever of this kind, in which, he says, the fever was rarely developed, if the diarrhea was judiciously treated. In an epidemic fever at Liverpool, England, in 1844, diarrhea was generally present. Cholera morbus, dysentery, diarrhea, and fever and ague are the forms of malarial disease that most frequently attack in winter. Cleghorn gives numerous tables of these facts, and universal experience corroborates them. The same testimony was often given in the *Medical Recorder*, published many years ago in this country.

It is evident, therefore, from all these facts, that the occasional attacks of cholera in winter constitute no reason for doubting the malarial origin of that disease. When properly understood, these developments of cholera in winter are excellent reasons for considering it a malarial disease; and a strong corroborative proof is found in the universal experience that cold weather checks all tendency to an epidemic prevalence of cholera. Its attacks are then scattered over a broad surface, and are far apart, just as we see fever and ague in cold weather.

A brief statement of the facts noticed on this subject, by medical men in various parts of the world must close this department of the subject. In Moscow, the disease prevailed and was most fatal, in a flat, malarious quarter, surrounded by a bend of the river Moskwa. At Breslau, cholera first attacked, and principally ravaged, that part of the town which is low and marshy, and which is the constant seat of intermittent fever. Dr. Antomachi says, that the condition of the houses in which cholera prevailed in Warsaw, was little better than that of sewers. In Paris, "the disease first appeared, and subsequently spread, above all other places, in the *quartiers* situated on the borders of the Seine; it was most prevalent and fatal in the low, close, undrained, and uncleansed localities."

Dr. Guy says, in his "Report on Public Health," from which we quote a portion of the above facts, that "in

England cholera, generally, first appeared in the neighborhood of marshes or rivers, and principally raged in low, damp localities, particularly where these were also the outlets of filth. In Carlisle, for example, it is stated that it first broke out near a mill, and raged down the dam-side; few cases occurring in any other part of the town." In Manchester the malarious localities exclusively suffered; we mean those parts near the *outlets* of drains, and in the immediate neighborhood of sewers. Open or obstructed sewers are fruitful sources of malaria, and these were the home of the cholera. The following singular fact was noticed at Liverpool, England:

"One morning it was discovered that several men had been seized with cholera, during the preceding night, on board a vessel lying in one of the docks. The men were sent to the hospital, and the vessel having been warped into the river, another ship with a healthy crew took up her station. The next morning all the hands on board were ill of cholera. On examining the dock, it was found that a large sewer discharged its contents under the spot where the vessel was placed."

The cholera cut off nearly half the inhabitants of Inver, a town situated on the low sandy shore of the Tain Frith, *and notorious for its malaria*, while the town of Tain and most of the rural districts escaped.

"All accounts from India agree in stating that cholera first breaks out and principally prevails in low and marshy situations, and particularly near the banks of rivers; that whenever a village or military station lies upon or near low, marshy, or damp ground, the occupants suffer in direct proportion to their proximity to such a situation; and that when a regiment has been encamped, one part on high and dry land and the other part on a morass, or on the bank of a river, it is constantly observed that the former has remained healthy, while the latter has suffered severely from the disease."

We have noticed many striking illustrations of these facts in military reports from India, Persia, and Russia. Dr. Guy says, very truly, "that in these respects cholera bears a close analogy to fever," and all readers of military medicine must be struck with this truth.

In London the cholera was confined to the old and familiar seats of malaria. The whole of the coast extending from St. Katharine's docks to Wapping, was frightfully ravaged by it. "It was the practice in that region to pump out the cellars to rid them of water which had got up into the houses by infiltration from the river, or more frequently flowing in through the house drains from the sewers when the tide forced back the water into the houses. The stench from the water pumped out of the cellars was often intolerable; so much so that I was accustomed to go out of the way to avoid it. Cess-pools were general, the contents of which percolated through the substratum, and the river water percolating through the substratum carried with it the matter from the cess-pools." This witness adds, as his opinion, "that cholera took the place of typhus, affecting the same class of persons, and being influenced by the same circumstances."

Of Southwark, Mr. Leadam says: "This was certainly one of the districts the most severely visited by cholera. The disease prevailed chiefly in the filthy dens which we have about us, in the close courts and alleys. The cholera and typhus track were identical." Mr. Wagstaffe, after describing the condition of Lambeth, says: "these localities, in which typhus is constantly present, are the very localities in which cholera chiefly raged. I have at the present moment many cases of fever in the very places in which cholera was most prevalent. This autumn, diarrhea and dysentery have also been prevalent there, and some cases were so similar to Asiatic cholera that I have asked some of my professional brethren to go and see them; two of these cases were fatal.

They had, in fact, all the characteristic symptoms—vomiting, diarrhea, with rice colored evacuations, cramps, suppression of rine, the particular sunken countenance, giving the expression of age to the patient, with a livid or even blue color."

"In the parish of Christ Church," says Mr. Doubleday, "and in the neighborhood of Broadwall, there are *open sewers*. At Brunswick Place there is an open one. In these neighborhoods the cholera was unusually severe; in one row of houses, within two yards of the sewer, houses which are very miserable as regards size, ventilation, and means of cleanliness, the mortality was excessive; as many as five died in one house."

And thus might we go on over the whole earth, and we should find in all cases of cholera traces of present malaria, or of its recent existence. We have traced its habitations and habitudes, and have been particular in details, in order to make the sanitary regulations we wish to urge, the more impressive from being founded on reason and knowledge. We feel the most perfect confidence in the fact that all measures that tend to prevent the formation of malaria will completely prevent the production of cholera, and all other malarial diseases.

The wide sweep of cholera over the earth has been attended with a great many advantages to humanity. Never in the world's history has the attention of men been so effectually and efficiently aroused as by the epidemic influences of cholera, and universal inquiry has resulted in the development of much precious truth, and of lasting benefit to mankind. Old opinions have been modified; new principles brought to light; and permanent good has been done by the enlightened action of medical men in their investigations of the condition of the cholera localities of Europe. The Reports of the Commissioners of Public Health, upon the condition of towns in

England, which have been made from year to year since cholera invaded that kingdom, contain a body of facts that must be of the highest importance to the public welfare. They show in the most impressive manner the direful effects of improper drainage, sewerage, etc., and the grave evils of bad ventilation. Defective drainage, and sewerage, coupled with want of ventilation, exhibit winter effects of malaria. Dr. Stark, of Edinburgh, points out these effects with great clearness, and his facts should be known to the public authorities of all large and growing towns. The wise and long-continued investigations of the medical men of England, into the condition of the various towns of the kingdom, and the history of disease in them, very fully corroborate the views and facts of Dr. Stark. They establish one material point very clearly, and that is that malaria may be so cooped up as to defy the sanative effects of cold weather. We referred to this valuable fact in our remarks upon malaria in the holds of ships, while winter had possession of the decks. Large, filthy sewers are to a neighborhood what the hold of a vessel is to the people on deck. Malaria cannot easily be formed in winter, upon an open marsh, but may in the hold of a ship, and in badly constructed sewers. Until the public mind was drawn to these things, few persons imagined how extensively disease prevails in England, and how constantly it is connected with malaria. But in these "Public Reports" to which we have referred, we often meet with such facts as these: "The fever is almost, if not altogether, identical with a fever not long ago extensively prevalent in the large towns of Scotland, from whence it probably passed into England." "It appears, from the statement o a trustworthy correspondent, that the disease first appeared at Hunemanby in August last, and prevaile l more especially amongst the population resident in badly constructed tenements, built upon soft ground badly drained, with neglected privies and drains behind. * * * At Reighton, a small village three miles from Hunemanby, there have been thirty cases. Here there is a nasty marsh, with a dirty, foul pond close by, fed from some farm-yards above with a quantity of offensive fluid. All the thirty cases of fever occurred in close vicinity to this pond or marsh, while the upper portion of the village has been totally exempt from the epidemic." There are few towns in England altogether exempt from malaria.

Here is an important fact that should arouse the attention of western farmers, for the farmers of Kentucky are negligent upon these points. Dr. Laycock says: "I believe that few farmers are aware that the plan adopted by many, of leaving their straw, mixed with the dung of their cattle, to rot and become manure in a yard close to their premises, is the surest method they could devise of predisposing themselves and their servants to suffer severely from such an epidemic as the one in question. Where you have a quantity of straw rotting you must necessarily have an evolution of noxious gases. Now in a farm-yard where straw and dung are decaying together, you have an ample source of miasm, even if situated in a high and dry situation; and if the drainage from such yards is allowed to run along in an open channel, or into open ditches near the dwellings, you have the sources of miasm still more widely extended."

The immense value of perfect drainage and cleanliness cannot be too constantly enforced upon the attention of men. These are the sources of health; a neglect of them is the certain precursor of sickness and misery. Nature has provided the humblest insect tribes, and all animals inferior to man, with means of cleanliness, that are most perfect in their adaptation to the end in view. A survey of these truths gives an impressive lesson upon the necessity and value of cleanliness, and man cannot neglect this great law of the animal economy without paying the penalty. We cannot dwell upon the facts in Natural History, to which we allude, but in a future article on sanitary measures, we shall endeavor to present our readers a number of very curious facts on this interesting subject.

